# Abnormal Outer Choroidal Vasculature in Amblyopia

**DOI:** 10.1155/2019/2097087

**Published:** 2019-01-10

**Authors:** Noriko Terada, Manabu Miyata, Yuki Muraoka, Masayuki Hata, Masahiro Fujimoto, Satoshi Yokota, Hideo Nakanishi, Kenji Suda, Munemitsu Yoshikawa, Sotaro Ooto, Hiroshi Ohtsuki, Akitaka Tsujikawa

**Affiliations:** ^1^Department of Ophthalmology and Visual Sciences, Kyoto University Graduate School of Medicine, Kyoto, Japan; ^2^Department of Ophthalmology, Okayama University Graduate School of Medicine, Dentistry and Pharmaceutical Sciences, Okayama, Japan

## Abstract

**Purpose:**

Several studies have indicated morphological changes in the choroid in amblyopia cases. This study investigates whether choroidal vasculature was different among amblyopic and fellow eyes in unilateral amblyopia patients and healthy eyes, using en face images acquired via swept-source optical coherence tomography (SS-OCT).

**Design:**

Prospective, observational case-control study.

**Methods:**

This study included 14 consecutive patients with unilateral amblyopia and 22 age- and axial length-matched healthy eyes. Using SS-OCT, we obtained en face images of choroidal vasculature midway through the subfoveal inner and total choroid, corresponding to the vasculature of the choriocapillaris and Sattler's layer (inner choroid) and Haller's layer (outer choroid), respectively. We analyzed the en face images of the inner and outer choroidal vascular areas in 3 × 3 mm squares adjusted from 6 × 6 mm squares, using Littmann's magnification correction, after binarization of the images as a portion of the whole area.

**Results:**

The outer choroidal vascular areas were larger in both amblyopic and fellow eyes than in healthy eyes (both *P* < 0.001), although there were no significant differences in inner (56.35 ± 2.46% and 56.27 ± 3.75%, respectively) or outer (61.49 ± 4.95% and 61.48 ± 3.73%, respectively) choroidal vascular area between amblyopic and fellow eyes (*P*=0.98  and  0.91, respectively). An outer choroidal vascular area of 59% was set as an appropriate cutoff value for distinguishing patients from controls.

**Conclusions:**

The outer choroidal vascular area was larger in both amblyopic eyes and fellow eyes compared to healthy eyes. Our findings may help clarify the etiology of amblyopia.

## 1. Introduction

Amblyopia is a disorder involving dysfunction in the processing of visual information [1] or a deficit in optotype acuity, with no detectable organic cause [2]. However, recent studies using optical coherence tomography (OCT) have shown that amblyopic eyes exhibit greater subfoveal choroidal thickness than their fellow and control eyes [3–6], although the findings are controversial because a magnification factor was not applied [7]. Furthermore, a previous study that used B-scan OCT images reported a higher choroidal luminal/stromal ratio in amblyopic eyes than in fellow and control eyes [8], implicating morphological differences in the choroid may be a factor in amblyopia.

Recently, Hirashima et al. reported a method for investigating the choroidal vascular area using swept-source OCT (SS-OCT) en face images by dividing the region into the inner and outer choroid [9], in accordance with a previous report [10]. This method enables visualization of choroidal vasculature at the same level below the retinal pigment epithelium (RPE). In contrast to B-scan images, en face images eliminate the necessity of segmenting the choroidal vascular area, because the vasculature runs parallel to the RPE; it is, therefore, possible to investigate the choroidal area in amblyopic eyes with greater accuracy.

An anatomical comparison of amblyopic eyes with their fellow eyes may not be optimal as such fellow eyes, even with normal vision, might not in fact be healthy eyes because important phenomena, such as the McGurk effect in which interactions between hearing and vision in influence speech perception, are weaker in monocular viewing for both the amblyopic eye and the fellow eye compared to healthy subjects [11]. Furthermore, all children with high functional risk factors for amblyopia such as anisometropia and strabismus do not develop amblyopia. Thus, we hypothesized that there are anatomical risk factors for the onset of amblyopia aside from functional risk factors. In this study, we investigated whether the inner and outer choroidal vasculature were different among amblyopic, fellow, and healthy eyes using en face SS-OCT images in order to test our hypothesis.

## 2. Materials and Methods

### 2.1. Ethics

This prospective, observational case-controlled study was approved by the ethics committee of the Kyoto University Graduate School of Medicine (Kyoto, Japan). All study protocols adhered to the tenets of the Declaration of Helsinki. The nature of the study and the possible risks and benefits of participation were explained to all study candidates. All subjects who agreed to participate provided verbal informed consent.

### 2.2. Participants

We recruited consecutive patients with amblyopia who visited the Department of Ophthalmology and Visual Science at the Kyoto University Graduate School of Medicine, between October 2015 and July 2016. All patients underwent a comprehensive ophthalmological examination that included autorefractometry, measurements of best-corrected visual acuity (VA) using a decimal VA chart (Landolt chart) and of axial length (AL) using an IOL Master (Carl Zeiss Meditec, Inc., Dublin, CA), indirect ophthalmoscopy, slit-lamp biomicroscopy, and SS-OCT (DRI OCT-1, Topcon Corp., Tokyo, Japan). A clinical diagnosis of amblyopia was made by specialists in this field based on findings of this comprehensive ophthalmologic examination and the patient's medical history. Healthy individuals with a VA ≥ 20/20 were recruited at the Kyoto University Graduate School of Medicine, and age- and AL-matched subjects were selected as controls.

The inclusion criteria were as follows: treatment-naïve, in-treatment, or posttreatment amblyopia; unilateral anisometropic, strabismic, or meridional amblyopia; and good SS-OCT image quality. The exclusion criteria were as follows: the presence of other ocular diseases, except for mild refractive errors; inadequate patient cooperation for measurement; and an AL ≥ 26.0 mm.

### 2.3. Image Acquisition via Swept-Source Optical Coherence Tomography

**Figure 1:**
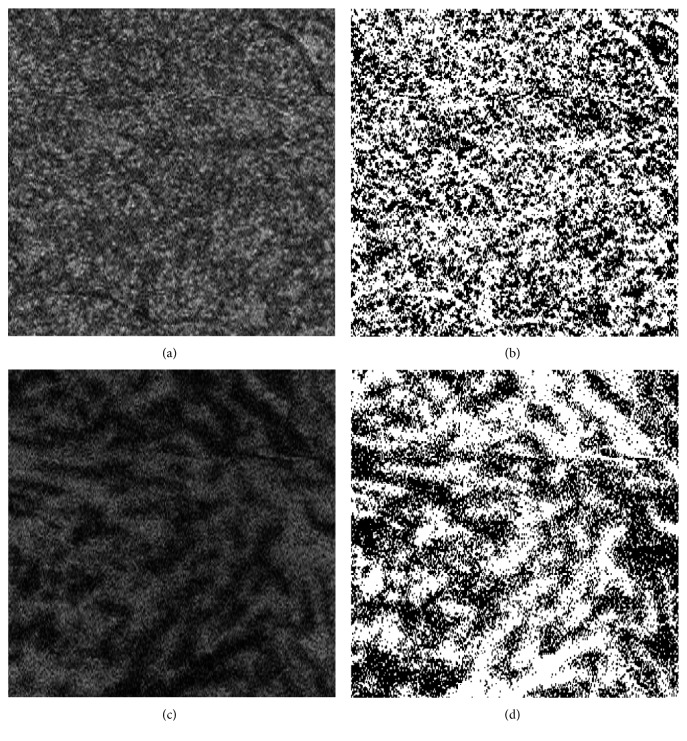
Representative choroidal en face and binarized images acquired at the level of the inner and outer choroid to measure the choroidal vascular area. Inner choroidal en face images of the right eye of a 5-year-old male patient with amblyopia before (a) and after (b) binarization. Outer choroidal en face images of the same eye before (c) and after (d) binarization. (a, c) White regions indicate the choroidal vascular area. (b, d) Black regions indicate the vascular wall and choroidal stroma.

Trained examiners performed SS-OCT evaluations after pupil dilation. During imaging, the examiners achieved pupil centration by using an internal fixation target that was confirmed using the built-in camera of the SS-OCT system. Each three-dimensional (3D) volumetric scan was centered on the fovea and covered an area of 6 × 6 mm, which consisted of 512 (horizontal) × 256 (vertical) A-scans. In each B-scan of the 3D data set, the outer surface of Bruch's membrane (BM) was automatically delineated by the software, and manual corrections were made as necessary using the built-in segmentation modifying tool. Using the software developed by Topcon Corp., en face images were automatically reconstructed from the 3D data set after flattening on the basis of the BM [10]. En face images of the choroid were acquired at varying depths, every 2.6 *μ*m, from the BM. These images showed the vascular area as white regions and the vascular wall and choroidal stroma as black regions (Figure 1).

### 2.4. Subfoveal Choroidal and Foveal Thickness Measurements

Horizontal B-scan SS-OCT images through the fovea were used to measure subfoveal choroidal and foveal thickness. Because these measurements have been shown to have high intraclass correlation [12], only one of the investigators (MM) manually measured these parameters using the built-in caliper tool. The outermost highly reflective retinal band comprises the RPE and BM [13]. Foveal thickness was defined as the distance between the vitreoretinal interface and inner border of the RPE-BM complex at the fovea. Subfoveal choroidal thickness was defined as the distance between the outer border of the RPE–BM complex and the chorioscleral interface at the subfovea. Furthermore, in accordance with previous reports [14, 15], the same investigator detected the border between the medium and large choroidal vessel layers and manually measured the subfoveal distance between the outer border of the RPE-BM complex and the border between the medium and large choroidal vessel layers, referring to horizontal B-scan images around the subfovea. This distance was defined as the subfoveal inner choroidal thickness, which corresponds to the thickness of the choriocapillaris and Sattler's layer (medium choroidal vessel layer).

### 2.5. Measurement of the Choroidal Vascular Area

To evaluate the choroidal vascular area, en face images were analyzed at the level of the inner and outer choroid. Each en face image was created by extracting data from 3D raster scans at 2.6 *μ*m increments in depth. These en face images were used for analyzing the choroidal vascular area midway through the subfoveal inner and total choroid, as described in previous reports [9, 10]. To correct for AL-related magnification, we derived an accurate 3 × 3 mm square en face image from an uncorrected default 6 × 6 mm square using Littmann's formula, which requires the values of AL, flatter and steeper meridians, and spherical equivalent refraction [16], because actual measurement squares are variable in each eye. The choroidal vascular area in the adjusted 3 × 3 mm image was automatically calculated using ImageJ software (National Institutes of Health, Bethesda, MD), as described previously [9, 10]. Briefly, vasculature and choroidal stroma were distinguished using the command path Image > Adjust > Threshold > Auto in ImageJ. Then, the chosen images were binarized using the Otsu method [17], which involves automatic threshold selection from grey-level histograms (Figure 1). Next, the area of the vascular lumen was calculated in pixels using the command path Analyze > Measure in ImageJ. In the present study, choroidal vascular area, expressed as a percentage, was defined as the portion of the vascular lumen in the whole scan area at the inner and outer choroid as previously reported [9].

### 2.6. Statistical Analysis

Data are presented as mean ± standard deviation where applicable. For statistical analysis, VA was converted to the logarithm of the minimal angle of resolution. Comparisons between amblyopic and fellow eyes in the patients and between both eyes in controls were performed using paired *t*-tests. Comparisons among amblyopic, fellow, and control eyes were performed using one-way analysis of variance, followed by a post hoc Tukey test or chi-square test where applicable. The cutoff value for the choroidal vascular area, the best sensitivity–specificity balance, and the area under the receiver operating characteristic curve (AUROC) were calculated. All statistical analyses were performed using SPSS version 23 (IBM Corp., Armonk, NY). *P* values < 0.05 were considered statistically significant.

## 3. Results

**Figure 2:**
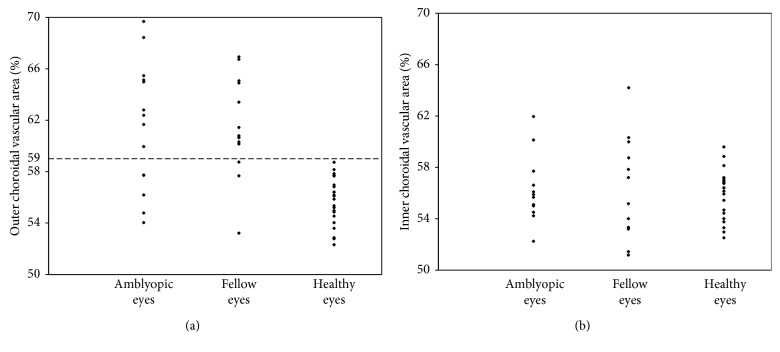
Scatter plot of the choroidal vascular area in amblyopic, fellow, and control eyes. (a) The outer choroidal vascular area both in amblyopic (61.49 ± 4.95%) and fellow (61.48 ± 3.73%) eyes is markedly larger than that in healthy eyes (55.69 ± 1.83%; both *P* < 0.001). When the cutoff value for the outer choroidal vascular area was set at 59%, we could distinguish between the amblyopic and healthy eyes with a specificity of 100% and sensitivity of 64% and between the fellow and healthy eyes with a specificity of 100% and sensitivity of 86%. (b) The inner choroidal vascular area did not differ among amblyopic (56.35 ± 2.46%), fellow (56.27 ± 3.75%), and healthy (55.73 ± 2.04%) eyes.

This study included 14 patients with amblyopia (age, 6.8 ± 3.2 years) and 22 healthy eyes of 11 controls (age, 7.1 ± 2.6 years); patient characteristics are provided in Table 1. Amblyopia was caused by meridian in 1, strabismus in 4, and anisometropia in 9 patients while 2 patients had treatment-naïve amblyopia, and 6 each had in-treatment and posttreatment amblyopia. Relative to fellow eyes, amblyopic eyes exhibited worse VA (*P*=0.02), lower AL (*P*=0.02) and greater subfoveal total and outer choroidal thickness values (*P*=0.03  and  0.04, respectively). However, there was no statistically significant difference in subfoveal inner choroidal thickness, foveal thickness, or choroidal vascular area between amblyopic and fellow eyes. There were no interocular differences in any parameters in control eyes. Among amblyopic, fellow, and healthy eyes, the outer choroidal vascular area in both amblyopic (61.49 ± 4.95%) and fellow (61.48 ± 3 .73%) eyes was markedly larger than in healthy eyes (55.69 ± 1.83%; both *P* < 0.001; Figure 2). There were no differences in inner choroidal vascular area among the eyes (amblyopic eyes, 56.35 ± 2.46%; fellow eyes, 56.27 ± 3.75%; healthy control eyes, 55.73 ± 2.04%). When the cutoff value for the outer choroidal vascular area was set at 59%, we could distinguish between the amblyopic and healthy eyes with a specificity of 100% and a sensitivity of 64%, and between the fellow and healthy eyes with a specificity of 100% and a sensitivity of 86%. For the AUROC, the best sensitivity-specificity balance achieved was 0.844 and 0.925, respectively.

## 4. Discussion

The present findings, derived by intrasubject analysis using en face SS-OCT images, demonstrated no significant differences in inner and outer choroidal vascular areas either between the 2 eyes in age- and AL-matched controls or between amblyopic and fellow eyes in the patients, although there were significant differences in VA, AL, and subfoveal choroidal thickness between them. However, the outer choroidal vascular area in both amblyopic and fellow eyes was markedly larger than that in healthy eyes. An outer choroidal vascular area >59%, even in fellow eyes with normal vision, may indicate a risk for amblyopia onset. This finding might be helpful in detecting amblyopia risk before onset and can arouse suspicion of amblyopia in cases where VA measurements are not easily obtained.

A previous retrospective study using spectral domain OCT B-scan images showed that there was an interocular difference in the luminal/stromal ratio in the choroid, corresponding to the choroidal vascular area in the present study, in patients with anisometropic amblyopia (*P*=0.04), and the ratio in amblyopic eyes was larger than that in control eyes (*P* < 0.001) [8]. However, the study did not reveal a difference in the ratio between fellow and healthy eyes. Our findings agree with the latter results, but not the former. Furthermore, we found that the outer choroidal vascular area in fellow eyes was markedly larger than that in healthy eyes. Therefore, we consider that fellow eyes, even with normal vision, may be anatomically abnormal and might affect the onset of amblyopia.

Amblyopia has been believed to be a neurodevelopmental disorder of the visual cortex [18]. However, Ikeda reported that retinal defects might cause amblyopia [19]. Considering the mechanism of increased outer choroidal vascular area without increased choroidal thickness, we hypothesized that less choroidal stroma might negatively affect visual development. Carotenoids, including lutein, have an antioxidant function [20] and play an important physiological role in protecting developing eye tissue from free radical damage. The presence of oxylutein is shown in the choroidal stroma throughout prenatal human development [21]. In the present study, choroidal stroma is decreased, contrary to the increased outer choroidal vasculature in the choroidal thickness similar to the healthy eyes. Therefore, a reduced antioxidant effect is likely to induce amblyopia; also, there might be a lack of other beneficial factors due to the decreased choroidal stroma.

**Figure 3:**
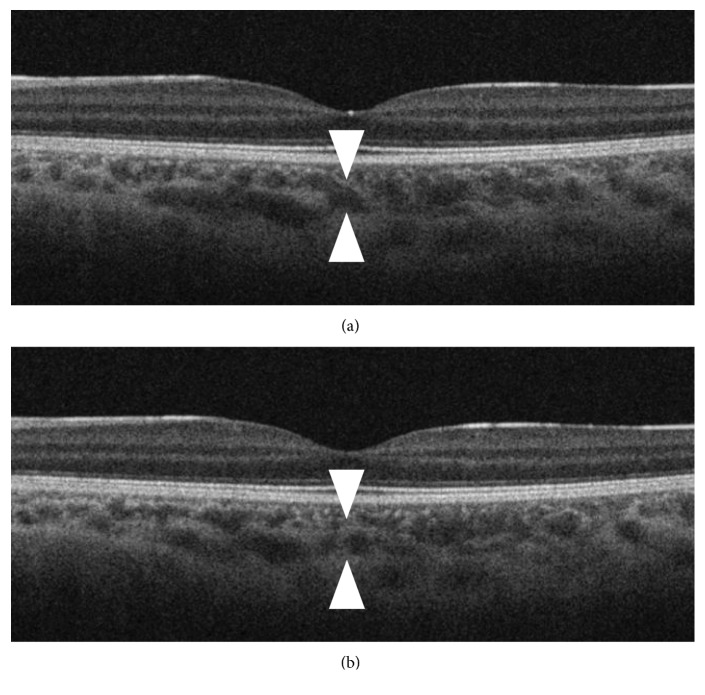
Differences in the cross section of choroidal vasculature in B-scan images. (a) A swept-source optical coherence tomography (SS-OCT) B-scan image through the fovea of the right eye of a 5-year-old male patient, along with the fellow eye. (b) SS-OCT B-scan image taken 3 slices away from that shown in (a). Cross sections of large choroidal vasculature varied substantially, even between neighboring slices (arrow heads indicate the same vessel).

In the present study, we used en face images, in accordance with previous reports [9, 10], to compare choroidal vascular areas. The advantage of this method is that it eliminates the necessity of segmenting the choroidal vasculature along its course. We consider this method to be superior to other methods that employ B-scan images because cross sections of large choroidal vasculature in B-scan images vary substantially, even between neighboring slices (Figure 3). The optimal method for such comparative analyses requires further investigation.

Many ophthalmologists have considered that there is no anatomical difference between amblyopic and fellow eyes. However, many recent reports, including the present study, have demonstrated greater choroidal thickness in amblyopic eyes than in fellow eyes [3–6]. This finding is reasonable because the AL of amblyopic eyes is smaller than that of fellow eyes in anisometropia cases; moreover, anisometropia is the most common cause of amblyopia. Furthermore, choroidal thickness is negatively correlated with AL in normal eyes [22]. Our results revealed no differences in choroidal vascular area between unilateral amblyopic eyes without organic disorders and fellow eyes. However, if unilateral amblyopic eyes did exhibit organic choroidal disorders, differences such as central serous chorioretinopathy would be noted between amblyopic and fellow eyes [10]. Therefore, intrasubject comparisons of choroidal vascular area in unilateral amblyopia would be helpful in excluding organic choroidal disorders.

Additionally, pachychoroid disorders are receiving a lot of attention in the field of retina research and imply choroidal manifestations, including increased choroidal thickness and dilation of the outer choroidal vessels, and are involved in the underlying pathology [23]. Pachychoroid features are sometimes associated with not only retinal diseases [24, 25] but also neuroophthalmological diseases including nonarteritic anterior ischemic optic neuropathy and glaucoma [26, 27]. In unilateral central serous chorioretinopathy, even the fellow eyes have pachychoroid features in 92% of cases [24]. In the present study, some of the patients with amblyopia had a much larger outer choroidal vascular area in both eyes. These patients may be prone to a spectrum of conditions characterized by pachychoroid features.

The present study had some limitations. First, the sample size was small. However, it is unlikely that including a larger sample would have produced different results, given that there was almost no difference between amblyopic and fellow eyes. Second, this study included patients in various stages of treatment. In many cases, we could not obtain clear en face SS-OCT images due to poor fixation ability. In the future, high-penetration OCT modalities with high scanning speeds would enable us to effectively evaluate more patients. Third, this study was cross-sectional in design. Choroidal thickness has previously been evaluated in a longitudinal study [3]; accordingly, a longitudinal study further analyzing changes in choroidal vasculature in amblyopia is desirable. Fourth, we could not assess the choriocapillaris monolayer because fine choriocapillaris structures could not be observed due to the limitation of current SS-OCT technology. Recently, OCT angiography was shown to detect fine choriocapillaris blood flow [28], although OCT angiography cannot detect large choroidal blood flow due to the rapid flow. A recent report using OCT angiography showed increased choriocapillaris vessel density in amblyopic eyes [29].

## 5. Conclusions

The outer choroidal vascular area was larger in both amblyopic and fellow eyes compared to healthy eyes. Our findings may help to clarify the etiology of amblyopia.

## Figures and Tables

**Table 1:** Characteristics of the study population.

Clinical parameters		Amblyopic eyes (1)	Fellow eyes (2)	Healthy eyes (3)	P^#^ (1) vs (2)	P^##^ (1) vs (3)	P^##^ (2) vs (3)
*N*	Anisometropia	9		22	—	—	—
	Strabismus	4			—	—	—
	Meridian	1			—	—	—
Age (years)		6.8 ± 3.2		7.1 ± 2.6	—	0.80^+^	
Female sex portion (%)		50		64	—	0.50^++^	
Right eye (*n* (%))		5 (36)		—	—	—	—
LogMAR VA		0.16 ± 0.40	−0.11 ± 0.09	≤0	0.02^*∗*^	—	—
Axial length (mm)		21.74 ± 1.10	22.15 ± 1.14	22.32 ± 0.74	0.02^*∗*^	0.21	0.87
Choroidal vascular area (%)	Inner	56.35 ± 2.46	56.27 ± 3.75	55.73 ± 2.04	0.98	0.79	0.83
	Outer	61.49 ± 4.95	61.48 ± 3.73	55.69 ± 1.83	0.91	<0.001^*∗*^	<0.001^*∗*^
Subfoveal choroidal thickness (*μ*m)	Total	375.4 ± 87.6	344.9 ± 94.4	351.9 ± 60.7	0.03^*∗*^	0.66	0.96
	Inner	94.5 ± 29.0	91.0 ± 24.5	91.9 ± 16.0	0.23	0.94	0.99
	Outer	280.9 ± 73.1	253.9 ± 82.9	260.0 ± 54.2	0.04^*∗*^	0.65	0.96
Foveal thickness (*μ*m)		183.0 ± 12.8	183.3 ± 13.8	178.8 ± 5.9	0.89	0.48	0.44

Data are presented as mean ± standard deviation where applicable. To measure the choroidal vascular area, one side of a 3 mm square was adjusted using Littmann's formula. logMAR, logarithm of the minimum angle of resolution; VA, visual acuity; ^#^paired *t*-test; ^##^Tukey test; ^+^unpaired *t*-test; ^++^chi-square test; ^*∗*^statistically significant (*P* < 0.05).

## Data Availability

The data used to support the findings of this study are included within the article.
